# Evaluation of pharmacy practice program in the 6-year pharmaceutical education curriculum in Japan: hospital pharmacy practice program

**DOI:** 10.1186/s40780-015-0019-2

**Published:** 2015-06-26

**Authors:** Miho Utsumi, Sachi Hirano, Yuki Fujii, Hiroshi Yamamoto

**Affiliations:** Faculty of Pharmaceutical Sciences, Kobe Gakuin University, 1-1-3 Minatojima, Chuo-ku, Kobe-shi, 650-8586 Hyogo Japan

**Keywords:** Pharmacy practice program, Program evaluation, Pharmaceutical education, Pharmacist education

## Abstract

**Background:**

The objective of this study was to clarify the state of national pharmacy practice program in the 6-year course of pharmaceutical education from the students’ point of view. We will suggest the points for improvement and issues of the current pharmacy practice programs to enhance the educational effects of the pharmacy practice program.

**Methods:**

The survey conducted from September 2011 to March 2012 (hereinafter referred to as “2011”) and from September 2012 to March 2013 (hereinafter referred to as “2012”) comprised 1,607 pharmacy students, who had completed the pharmacy practice program. They were asked to fill out a self-descriptive questionnaire for the purpose of investigating the content of the pharmacy practice that the students themselves experienced, guidance provided by the supervising pharmacists, and support by the university faculty staff.

**Results:**

In order to clarify the factor structure of the overall results, four factors were extracted through an exploratory analysis: “satisfactory learning”, “support system of the training site (hospital)”, “support system of university”, and “dialogue with patients”. When we compared the score for each four factors between 2011 and 2012 and we found that 2012 was evaluated as significantly higher for all factors. Furthermore, opportunities for discussion and reflection with the students led to observation that 2012 exhibited significantly better results than 2011.

**Conclusions:**

The students evaluations for the quality of hospital pharmacy practice have improved in 2012 compared to evaluations in 2011. Regarding the four factors of “satisfactory learning”, “support system of the training site”, “support system of university”, and “dialogue with patients”, significant differences in the results from 2011 and 2012 were observed, indicating their marked improvement.

## Background

In recent years, the medical system in Japan has experienced various problems; such as the progression of a rapidly aging society with fewer children, increase and intensifying of patients’ needs for medical services, and shortage of doctors and nurses [1, 2]. Along with these sophisticated and complicated changes in the medical field, capabilities and performances required of pharmacists have been diversifying and increasing every year. However, the Japanese traditional pharmaceutical education focuses on the basic education to foster drug-discovery researchers, and therefore, more practical and more patient-oriented education to meet the previously-mentioned social needs is insufficient [3–5]. Thus, pharmacists are far from contributing as medical staff members in clinical practice. To overcome this problem, the discussion on how to improve pharmaceutical education was initiated. Consequently, it was decided to extend the 4-year long pharmacist training course in Japan to a 6-year long course and to introduce the following three items: (1) model core curriculums (in model core curriculums, they have set up common national learning objectives that are to be learned before graduation, at least, which accounts for 70 % of the curriculum of each university. The remaining 30 % is the original curriculum of each university), (2) 5-month pharmacy practice, and (3) Pharmaceutical Common Achievement Test (CBT: Computer-Based Testing and OSCE: Objective Structured Clinical Examination). The Fundamental Law of Education and the Pharmacists Act was amended in June 2004 and the pharmacist training course in Japan has been modified from the 4-year course to the 6-year course since 2006.

Accordingly, “Model Core Curriculum of Pharmaceutical Education” and “Model Core Curriculum for Pharmacy Practice” were presented in August 2002 and October 2003, respectively [6]. Upon the creation of these model core curriculums, they were analyzed with respect to the global pharmaceutical education standards and the capabilities that are required of pharmacists were considered [3, 4, 7–9]. Moreover, it was clarified that education should be directed towards training “the leaders of medical service” who are equipped with the professionalism as health professionals, and who have a complete understanding of pathology and diseases, and can contribute to the rational and effective pharmacotherapy, and patient care in order to meet the social needs [5, 10]. Furthermore, radical educational reforms were introduced. For example, a total of 1,446 items of the learning objectives that were categorized into knowledge, skills, and attitude, were presented while recognizing usefulness of the spiral learning of knowledge, skills, and attitudes advocated by Bruner, and a learners-centered education was adopted [11]. Above all, the implementation of the pharmacy practice (the traditional 4-week observational learning was reorganized into the 22-week participatory practice consisting of an 11-week hospital training and an 11-week community pharmacy training program upon the transition to the 6-year education system) was considered the key to the success of the 6-year pharmaceutical education (the key to the training of pharmacists who can contribute to medical service) and many discussions have been held for its introduction by the Committee of Pharmacy Education Reform of the Pharmaceutical Society of Japan and other committees [12].

Each university is required to conduct a pre-clinical training of pharmacy practice for the 4^th^-grade pharmacy students. The students are required to pass Pharmaceutical Common Achievement Test (CBT, OSCE) before attending the pharmacy practice, in order to prevent students without a pharmacist license from attending the pharmaceutical practice program at the training site. By doing so, each university guarantees high quality of students in the 5^th^ grade and onwards at the pharmacy practice site [12, 13].

Additionally, it was also decided that about 300 thousand yen of training fee per student (per one facility) should be paid from each university to the training hospital or the pharmacy.

In the previous system, the 4-year curriculum pharmacist instructors had few opportunities to learn how to teach pharmacy students. Thus, previous pharmacy practice course had been entrusted only to the capability or instruction ability of the individual pharmacist instructors. Since 1999, however, Pharmacist Training Workshop for Pharmacy practice Instructor (i.e., previous Workshop for Pharmacy Educator) sponsored by Council on Pharmaceutical Education (sponsored by Japan Pharmacists Education Center from 2005 to 2010) has been held in various places in Japan. This has also led to the education of pharmacist instructors by clinical pharmacists, who have sufficient training experience of university teachers and young pharmacists. In the Pharmacist Training Workshop for Pharmacy practice Instructor, a program is available to develop clinical pharmacists’ interest in education and cultivate ability to design a curriculum for the purpose of “bringing about valuable changes in the learners’ behaviors (knowledge, skills, and attitudes)”. As a result, approximately 20,000 pharmacy practice instructors have been certified to date [14].

To ensure that these pharmacy practice programs are of a high quality, many studies have been conducted in foreign countries as well with regard to the training contents, instruction methods, to evaluate the students’ readiness for pharmacy practice, and the evaluation method for the pharmacy practices [15–18]. In addition, examinations are being conducted with regard to the ability of pharmacist instructors who are in charge of teaching and managing pharmacy practice, and likewise, pharmacist instructors are evaluated from a student’s point of view [19, 20]. There are also other examinations that investigate the quality of the students’ practical pharmacy work at their training site [21, 22]. The learning goals of the Model Core Curriculum for Pharmacy practice in our country have been set with the concept that “Team Medical Care” and “Community-Based Care” should be learned in the hospital pharmacy practice and the community pharmacy practice, respectively. Additionally, some individually case reports about pharmacy practice program in each facility and in each area have already been in existence. However, the current status of the nationwide pharmacy practice, national trends, and competency that pharmacy students should obtain with an eye on Japan’s today and future are not yet clarified. Moreover, there is no performance index to assess the pharmacy practice program in Japan.

In this study, therefore, we will clarify the current situation and problems faced in contemporary pharmacy practice to enhance the educational effects of the pharmacy practice program that started since the introduction of the 6-year pharmaceutical education. In the process, we will extract the performance index for the pharmacy practice program and perceive the whole picture and the national trends of the pharmacy practice program with the performance index. With an aim to provide support for nurturing highly skilled pharmacists that can contribute to the diversifying patient care, we will also discuss the ideal characteristics of effective pharmacy practice in line with the Japan’s actual pharmaceutical situation.

Note that the discussion in this study is limited to the hospital pharmacy practice in Japan; community pharmacy practice will be discussed in a separate paper.

## Methods

This study was conducted from September 2011 to March 2012 (hereafter referred to as “2011”) and September 2012 to March 2013 (hereafter referred to as “2012”). The participants were 1,607 pharmacy students in their 5^th^ and 6^th^ year (7.9 % of the combined total of 20,389 pharmacy students in 2010 and 2011), who had completed the pharmacy practice programs (hospital pharmacy practice and community pharmacy practice) during 2010 and 2011. For extracting target students, convenience sampling was employed to identify respondent schools so that we would receive responses from across the nation in view of clarifying the current status of the pharmacy practices conducted nationwide.

In addition to basic attributes of respondents, the questionnaire consisted of the following 48 items: 31 items on a 6-point scale, 15 items on a 2-point scale, 1 item of multiple choice question, and 1 item of free description questions. The questionnaire included items related the pharmacy practice content based on the students’ actual experience such as “A daily training schedule chart was prepared and pre-noticed” and “The actual training content was in line with the learning objectives”. There were also items related the learning environment such as “I felt that there were too many tasks that would not directly lead to my learning as a trainee” and “When screening of prescriptions or dispensing, two pharmacists were involved to ensure the safety”. Moreover, the items that seemed to contribute to deepening of the learning were included; namely, “Did you have a chance to have discussions/reflection sessions among students”? and “Did you have a chance to visit other facilities to learn advanced contents such as the emergency area, the perinatal area, and the health and welfare area for the aged?” Furthermore, we made an inquiry regarding the occurrence of problems at the training site by asking “Did you get into any trouble with a patient?” and regarding the training status of the pharmacist instructors by asking “The pharmacist (who was involved in your training) worked very hard in coaching you” and “The pharmacist was well prepared for your questions”. Another item was regarding the support status of the university teachers was included by asking “The university teachers provided you enough feedbacks on your learnings at your training site”.

Rx64 (version 3.1.0), js-STAR (release 2.0.6j), and Excel (2007) were used for statistical analysis. The average value of the corresponding item was assigned to the missing value of the response on a 6-point scale, and the missing value of the response on a 2-point scale was excluded from the analysis. To clarify the factorial structure of the entire responses, an exploratory factor analysis was performed for 31 items on a 6-point scale (with maximum likelihood estimation and promax rotation). Homogeneity of variance of factors extracted by factor analysis for 2011 and 2012 were investigated by using the Levene test. Consequently, Welch’s t-test was used for the comparison between the two years because an unequal variance was observed in some factors. A simple tabulation and chi-square test were conducted for the items on a 2-point scale to compare the difference between the two years.

This study was approved by the institutional review board　of Kobe Gakuin University. Then, we described that there is absolutely no influence on the academic record without participation of the survey.

## Results

### Attributes of respondents

There were a total of 1,410 respondents of the questionnaire (the valid response rate: 87.7 %) and the average age of the respondents was 24.1 ± 1.5 years old (in 2011: 24.2 ± 1.8 years old, in 2012: 24.1 ± 1.4 years old). Table 1 shows the details of the attributes by year Note that the responses that failed to fill in the basic attributes (age, sex) and failed to address three or more items were excluded from the target population.

**Table 1:** Attributes of the respondents

		Respondents (%)	
		2011	2012
Sex			
	Male	212 (42)	376 (41)
	Female	290 (58)	532 (59)
Area			
	Hokkaido	―	―
	Tohoku	―	―
	Kanto	134 (27)	568 (62)
	Hokuriku	―	―
	Tokai	20 (4)	0 (0)
	Kinki	196 (39)	89 (10)
	Chugoku/Shikoku	31 (6)	114 (13)
	Kyusyu/Yamaguchi	121 (24)	137 (15)
		502 (100)	908 (100)

### Factor analysis

Thirty one items on a 6-point scale were analyzed by factor analysis using the maximum likelihood method and promax rotation. On the basis of the change of the magnitude of eigenvalues and the variation as factors, 4-factor solutions were determined as most appropriate in terms of the number of factors. We repeated factor analysis using the combined data of 2011 and 2012 while excluding the items that indicated the ceiling effect and floor effect, those that showed factor loadings of 0.35 or more in the multiple factors, and those that did not show factor loadings of 0.35 or more in any factors. As a result, 19 items, a total of four factors, were selected (Table 2). The inter-factor correlations among the four factors were found to be as follows: r = 0.67 (between the 1^st^ factor and the 2^nd^ factor); r = 0.40 (between the 1^st^ factor and the 3^rd^ factor); r = 0.53 (between the 1^st^ factor and the 4^th^ factor); r = 0.30 (between the 2^nd^ factor and the 3^rd^ factor); r = 0.49 (between the 2^nd^ factor and the 4^th^ factor); and r = 0.28 (between the 3^rd^ factor and the 4^th^ factor). Thus, the highest level of correlation was found between the 1^st^ factor and the 2^nd^ factor. The Cronbach’s alpha between the items constituting the factors was also calculated by each factor to examine the internal consistency of the scale. Consequently, relatively high coefficient alphas were confirmed as follows and the credibility of each of them was confirmed: α = 0.76 (the 1^st^ factor), α = 0.75 (the 2^nd^ factor), α = 0.72 (the 3^rd^ factor), and α = 0.69 (the 4^th^ factor).　As this research is an exploratory pilot study, we retained the interpretive notions in our observation and thus decided to a slightly lower significance standard than usual (factor loading >0.40, α >0.70).

**Table 2:** Results of the factor analysis of evaluation items (by 6-point scale) of the students’ pharmacy practice

Question	FA1	FA2	FA3	FA4
(1) I am satisfied because I was able to learn what would be useful for my career as a pharmacist.	**0.84**	0.02	−0.05	−0.05
(2) I was able to experience the overall work of a pharmacist sufficiently.	**0.84**	−0.09	−0.05	0.01
(3) The actual learning contents were in line with the learning objectives (SBOs).	**0.72**	−0.08	0.03	−0.01
(4) I felt that I had too many tasks that would not lead to learning for me as a trainee.	**−0.65**	−0.01	0.05	−0.01
(5) I was able to experience “team medical care” at hospital sufficiently.	**0.63**	0.02	−0.03	0.06
(6) Some learning contents were far from pharmacist’s work.	**−0.52**	0.03	0.05	0.03
(7) Training schedule by hour was prepared and informed to me in advance.	**0.49**	−0.05	0.03	−0.06
(8) At the beginning of the training, an appropriate orientation useful for the training was conducted (e.g., briefing on the overall work of hospital and pharmacy).	**0.46**	−0.10	0.19	0.02
(9) I felt a gap between the ideal and real work of a pharmacist.	**−0.43**	−0.01	0.13	0.02
(10) I think I can work in clinical practice without problem because I was able to obtain a better understanding of the role of medicine or a pharmacist to some extent.	**0.36**	0.12	0.00	0.04
(11) The pharmacists facilitated your appropriate self-learning so that you can solve the problems of the patients.	**0.35**	0.34	0.04	0.05
(12) The pharmacists were able to have an empathetic communication with you.	−0.09	**0.95**	0.02	0.00
(13) The pharmacists accepted you as a member of the team.	0.05	**0.72**	0.08	−0.03
(14) I experienced some problems getting along with people at the training site.	−0.14	**−0.46**	0.11	0.04
(15) The university teachers gave you enough feedback on your learnings at the training site.	−0.15	0.07	**0.91**	0.00
(16) The university teachers provided enough support for me so that I can prevent trouble or work on the practical training smoothly in case of trouble.	0.01	−0.02	**0.85**	−0.02
(17) The practical training contents were well matched with the classes from the 1st to the 4th years (excluding the pre-practice training).	0.24	−0.03	**0.36**	−0.03
(18) I was able to have enough time with patients to talk with them.	−0.02	−0.01	−0.02	**1.02**
(19) I was able to have enough time with patients to explain about drugs or the disease.	0.07	0.02	0.03	**0.75**
Inter-factor correlations				
FA1		0.67	0.40	0.53
FA2			0.30	0.49
FA3				0.28
Cronbach’s alpha	0.76	0.75	0.72	0.69

The factors were defined as following: the 1st factor (FA1) is “satisfactory learning (hospital);” the 2nd factor (FA2) is the “support system of the training site (hospital);” the 3rd factor (FA3) is the “support system of the university;” and the 4th factor (FA4) is “dialogue with patients.” The bold numbers are the factors loadings that were considered significant

Each factor was interpreted as follows: the 1^st^ factor consisted of 11 items including “I am satisfied because I was able to learn what seemed to be beneficial for me working as a pharmacist” and “I was able to experience pharmacist’s work sufficiently”. It was named as “satisfactory learning” because it was interpreted as representing the students’ sense of satisfaction and fulfillment for pharmacy practice. The 2^nd^ factor consisted of three items including “The pharmacist was able to have empathetic communication with you” and “I experienced some problems getting along with people at the training site”. It was named as “support system of the training site” because it was interpreted as representing the system for accepting the students at the training site. The 3^rd^ factor consisted of 3 items including “University teachers provided me enough support to facilitate the pharmacy practice programs to prevent troubles beforehand or at the time of trouble” and “The pharmacy practice contents were well matched with the curriculum from the 1^st^ to the 4^th^ years (excluding the pre-clinical training)”. It was named as “support system of university” because it was interpreted as representing the students’ support system in pharmacy practice by university teachers and the model core curriculum based on learning of the contents from the 1^st^ to 4^th^ grade at university. Lastly, the 4^th^ factor consisted of 2 items including “I was able to have enough time to listen to patients” and “I was able to have enough time to explain to patients about drug therapies and diseases”. It was named as “dialogue with patients” because it was interpreted as representing the relationship with patients.

Next, the scores of the four factors in 2011 and in 2012 were calculated. The following are the details of each score; “satisfactory learning” (the mean ± SD in 2011: 3.930 ± 0.531, the mean ± SD in 2012: 4.033 ± 0.508), “support system of the training site (the mean ± SD in 2011: 3.777 ± 0.700, the mean ± SD in 2012: 3.910 ± 0.722), “support system of university” (the mean ± SD in 2011: 3.706 ± 1.126, the mean ± SD in 2012: 4.095 ± 1.010), and “dialogue with patients” (the mean ± SD in 2011: 4.333 ± 1.297, the mean ± SD in 2012: 4.496 ± 1.204), the average difference of all factors (the mean ± SD in 2011: 15.75 ± 2.604, the mean ± SD in 2012: 16.53 ± 2.383). We compared the scores for each of the four factors between 2011 and 2012. Consequently, for each factor, as well as on the whole, the score was significantly higher in 2012 than in 2011. The following are the details of each factor: “satisfactory learning” (t = −3.562; df = 995; *p* < 0.001; 95 % CI:-0.161 to −0.047), “support system of the training site (t = −3.358; df = 1060.691; *p* < 0.001; 95 % CI: −0.209 to −0.055), “support system of university” (t = −6.445; df = 943.182; *p* < 0.001; 95 % CI: −0.508 to −0.271), and “dialogue with patients” (t = −2.324; df = 970.725; *p* < 0.05; 95 % CI: −0.301 to −0.025). With regard to all factors, the average difference value was significantly higher in 2012 (t = −5.607; df = 958.962; *p* < 0.001; 95 % CI: −1.064 to −0.512).

### Simple tabulation and the chi-square test

Comparison of the binary data in 2011 and 2012 were performed using a simple tabulation and the chi-square test to assess the qualitative aspect of the pharmacy practice. It was observed that the patient assignment system was introduced more significantly in 2012 than in 2011 (*p* < 0.01). Further, the opportunity of having discussions/reflection sessions among students at the training site as well as at other visiting facilities increased significantly (*p* < 0.01 for both items) (Table 3). However, the opportunity for the students to make a presentation among pharmacists at an occasion such as a conference decreased more significantly in 2012 than in 2011 (*p* < 0.01).

**Table 3:** Results of the simple tabulation and the chi-square test of evaluation items (by 2-point scale) of the students’ pharmacy practice

Question		2011	2012	χ^2^ (1)	*P*
(1) Was a patient assignment system involved in the drug administration guidance which enabled you to take care of the same patients every time?	Yes	236 (49)	521 (59)	13.612	<0.01
	No	247 (51)	356 (41)		
(2) Did you have a time to have a discussion or a review among students at the training site?	Yes	254 (51)	581 (64)	24.198	<0.01
	No	248 (49)	324 (36)		
(3) Did you have opportunity to associate with other professionals (doctors, nurses, and others) to have discussions (including all the occasions such as conversation over the phone, communication with the medical team, or a casual daily talk in a ward)?	Yes	373 (75)	632 (70)	3.362	0.07, n.s.
	No	127 (25)	273 (30)		
(4) Did you have opportunity to associate with other students (medical students, nursing students, and others) to have discussions?	Yes	83 (17)	135 (15)	0.565	0.45, n.s.
	No	419 (83)	773 (85)		
(5) Did you have any opportunity to have a conference among pharmacists?	Yes	331 (67)	571 (64)	1.087	0.30, n.s.
	No	166 (33)	326 (36)		
(6) Did you have any opportunity to make a presentation at a conference among pharmacists?	Yes	156 (31)	222 (25)	7.071	<0.01
	No	343 (69)	683 (75)		
(7) Did you have any opportunity to visit other facilities?	Yes	133 (27)	353 (39)	21.275	<0.01
	No	366 (73)	551 (61)		
(8) Did you pay any training expense or training cost when you attended a practical work or training at the outside of the training site?	Yes	28 (6)	98 (11)	10.285	<0.01
	No	474 (94)	807 (89)		
(9) Did you have a time when you felt that you were forced to do simple tasks repeatedly?	Yes	176 (35)	273 (30)	3.288	0.07, n.s.
	No	326 (65)	631 (70)		
(10) Did you have any trouble with a patient?	Yes	4 (1)	14 (2)	0.898	0.34, n.s.
	No	498 (99)	893 (98)		
(11) Did you have any trouble with your pharmacist instructor?	Yes	26 (5)	50 (6)	0.023	0.88, n.s.
	No	476 (95)	855 (94)		
(12) Did you have any trouble among students?	Yes	7 (1)	20 (2)	0.740	0.39, n.s.
	No	495 (99)	887 (98)		
(13) Did you have any unreasonable occasions that you felt you got a tantrum from a pharmacist or were put the responsibility of a preparation error upon you during the training?	Yes	44 (9)	72 (8)	0.178	0.67, n.s.
	No	458 (91)	832 (92)		
(14) Did the hospital pharmacy practice program have an influence on your future career?	Yes	361 (72)	631 (71)	0.334	0.56, n.s.
	No	139 (28)	263 (29)		

Only approximately 70 % of all students had an opportunity to associate and have a discussion with other staff (doctors, nurses, and others) and there was no significant difference between the two years. It was also indicated that problems which caused discrepancy in opinions or a breach of trust in relationships occurred each year at a certain rate for patients, pharmacists, and other trainees (approximately. 1 %, 5 %, and 1 %, respectively). In addition, approximately 30 % of all students answered that they had an experience that made them feel they were forced to do non-academic and non-technical simple tasks such as replenishing drugs or sorting out prescriptions repeatedly. It was also shown that approximately 10 % of all students felt that they had to face outbursts from the pharmacist or were unjustly held responsible for a preparation error during training. It was also found out that the students had to pay their own training expenses (admission fees and transportation fees) for participating training programs provided by a local pharmaceutical association, significantly often in 2012 (*p* < 0.01).

Lastly, approximately 70 % of all students answered that the hospital pharmacy practice had an influence on the selection of their future course (e.g., “The hospital pharmacy practice made me decide to pursue my career as a hospital pharmacist”, “In the hospital pharmacy practice I was able to find my ideal pharmacist”, “I felt satisfied with the work at hospital”, and “I found I am not cut out to be a hospital pharmacist”.). There was no significant difference among these items between the two years.

Note that students who had an opportunity to have discussions/reflection sessions among other students as the follow-up measures provided by their universities (i.e., in the seminar or post-pharmacy practice poster presentations or briefing sessions) accounted for approximately 80 % of all, and there was no significant difference between the two years (X^2^(1) = 1.465, p = n.s.(0.23)).

### Multiple choice question

The actually services of pharmacist in each training site were revealed by multiple choice question (“Have you ever seen any of services of pharmacist in training site during training?”). It was found that 90 % or more of all students see that most the services of pharmacist are related to Model Core Curriculum for Pharmacy Practice were actually carried out. On the other hand, it was also shown that less than 90 % of all students see that the services for medication therapy management, preventing medical accidents, therapeutic drug monitoring, and critical care for the poisoned patient were carried out (approximately 68 %, 89 %, 75 % and 42 %, respectively)

### Free description

The following opinions were also comparatively frequent among students: “the training content and environment should be standardized so that there is no disparity for students or facilities”, “training was an important experience that I could not have had in the university”, “it was a good opportunity to consider my vision of the future”, “there were more classroom learning time at hospitals; thus, I was unable to participate in ward duties and team medicine as much”, etc.

## Discussion

From the result of the exploratory factor analysis performed for 31 items on a 6-point scale, it was indicated that the students evaluations on the hospital pharmacy practice program have improved in 2012 compared to evaluations in 2011. In particular, it was revealed that factors such as “satisfactory learning”, “support system of the training site”, and “support system of university” have improved significantly. In addition, as “support system of the training site” had a great influence on whether or not the students could experience the pharmacist work sufficiently and achieve “satisfactory learning” (inter-factor correlation = 0.67), it was considered necessary that the maximum continuous support from the university should be provided depending on each situation and considering the conditions of the training site, the pharmacist instructors’ qualification level, and the training system. An opportunity of “dialogue with patients” had a great influence on whether or not the “satisfactory learning” could be achieved (inter-factor correlation = 0.53). This created an opportunity in which students can have a dialogue with a patient through the drug administration guidance, and feel a sense of satisfaction and fulfillment as pharmacists and in order to obtain a deeper learning was suggested to be important.

When the results were reviewed from the perspective of the above mentioned methods and the 15 items on a 2-point scale for simple tabulation and the chi-square test, it was indicated that the number of facilities that utilize the patient assignment system, which ensures the same patient is assigned every time at the drug administration guidance, are increasing significantly. Facilities that allow the students to have a discussion on the cases among themselves and review their involvement in the patients and treatment, are also increasing significantly. It was inferred that these efforts by the pharmacist instructors of the facility contributed to the students’ sense of satisfaction and fulfillment of the objective of the pharmacy practice program. It was also revealed that students’ opportunities to visit other facilities significantly increased, which suggested that the pharmacist instructors in each facility devised a way to achieve all learning objectives of Model Core Curriculum for Pharmacy Practice.

Furthermore, although there was no difference regarding the opportunity to present in a conference on improvement of patient care or pharmacy service among pharmacists between 2011 and 2012, the students’ opportunities to give a presentation decreased significantly. Approximately 30 % of all students felt that they were forced to do simple tasks repeatedly and approximately 10 % of them answered that they had faced outbursts from the pharmacist or were unjustly held responsible for a preparation error during training. A possible potential factor explaining such occurrences is the additional workload and the burden experienced by the pharmacists as a result of accepting the trainees.

In order to turn the pharmacy practice into a profound learning opportunity, it is necessary to clarify the capabilities and qualifications required for the pharmacist instructors and to improve “the content of Pharmacist Training Workshop for Pharmacy Practice Instructor” and to deepen and improve the understanding of student education. Further, establishing and strengthening learning system parameters, such as the hospital size, the ratio of pharmacist instructors vs. students, and the coaching/intervention by university teachers taking into consideration the workload and fatigue of the pharmacist instructors, might be necessary.

The students who had an opportunity to have discussions/reflections among other students back in their universities (i.e., in a seminar or post-pharmacy practice poster presentations or briefing sessions) accounted for only about 80 % of all, which suggested approximately 20 % of the students did not have sufficient opportunities that could lead to the deepening of learning at each university.

With regards to the pharmacy practice, the students who had an opportunity to associate and have a discussion (including all occasions such as over the phone, with the medical team, and through a casual talk in a ward) with the other staff (doctors, nurses, and others) accounted for only about 70 % of all, albeit with “Team Medical Care” being as the primary objective of the training. There was no significant difference in the above items between 2011 and 2012, which indicated a situation that involved several causes at a certain rate and prohibited communication with other staff members who were part of the team medical care; these causes are as follows: the trainees originally did not have sufficient education and knowledge as pharmacists and did not have enough communication skills; the work contents for the pharmacist at the training site did not correspond to the team medical care; the pharmacist instructors were too busy. One factor behind this might be that the awareness that the inter-professional work (IPW) is important was not widespread in every clinical practice in Japan, which contributed to the failure of sufficiently fostering the medical environment and culture as well as inter-professional mutual understanding to put a team of medical care students into practice. The fact that the number of university that is working on the inter-professional education (IPE) to put an effective IPW into practice is still small was also considered as one factor. Consequently, it was considered that some of the pharmacists in clinical practice could not fully participate in the team medical care and therefore it was inferred that students got into the situation where they themselves could not learn “Team Medical Care” in the pharmacy practice. As a supplementary or remedial measure for these situations, it might be possible to provide students with opportunities to associate with other professionals in order to make them understand the importance of the IPW and to implement the IPE in the classes or the pre-clinical training during from the 1^st^ to the 4^th^ year. Additionally, the situation might also improve by explaining the purpose of the pharmacy practice program in the 6-year pharmaceutical education system as well as the purpose of accepting the trainees or by asking other medical professionals for cooperation for the training.

Considering approximately 70 % of all students answered that the hospital pharmacy practice program had an influence on their future career decisions, it was considered that letting pharmacy students, who are responsible for the next generation of medicine, experience the actual success and satisfaction through the participatory pharmacy practice program could lead to the improvement of their motivation to contribute to medical care and society. Therefore, cooperation by the current pharmacists, other health professionals, university teachers, and community people to work on the pharmacy practice to foster high-quality pharmacists might also lead to the improvement of the quality of medical care.

## Conclusions

It was indicated that the students’ evaluation of the pharmacy practice program conducted in our country since the introduction of the 6-year pharmaceutical education system has been improving year after year. It was also revealed that whether “satisfactory learning” can be achieved from the pharmacy practice program or not is strongly influenced by “support system of the training site” and “dialogue with patients”. It was also considered that “dialogue with patients” is associated with the students’ sense of achievement and satisfaction. It is considered important that the training site, university, and community should be all unified as a whole to work on the student education for the purpose of fostering excellent pharmacists who can contribute to medicine.
